# Correction: Reinforcing biofiller “Lignin” for high performance green natural rubber nanocomposites

**DOI:** 10.1039/d5ra90127h

**Published:** 2025-11-07

**Authors:** Yuko Ikeda, Treethip Phakkeeree, Preeyanuch Junkong, Hiroyuki Yokohama, Pranee Phinyocheep, Ritsuko Kitano, Atsushi Kato

**Affiliations:** a Faculty of Molecular Chemistry and Engineering, Kyoto Institute of Technology Matsugasaki, Sakyo Kyoto 606-8585 Japan yuko@kit.ac.jp; b Graduate School of Science and Technology, Kyoto Institute of Technology Matsugasaki, Sakyo Kyoto 606-8585 Japan; c Department of Chemistry, Faculty of Science, Mahidol University Rama VI Road, Ratchthewee Bangkok 10400 Thailand; d NISSAN ARC, LTD. Natsushima-cho 1 Yokosuka Kanagawa 237-0061 Japan

## Abstract

Correction for ‘Reinforcing biofiller “Lignin” for high performance green natural rubber nanocomposites’ by Yuko Ikeda *et al.*, *RSC Adv.*, 2017, **7**, 5222–5231, https://doi.org/10.1039/C6RA26359C.

The ORCiD previously listed for Yuko Ikeda was incorrect. The correct ORCiD is: 0000-0003-2809-8911.

The Royal Society of Chemistry apologises for these errors and any consequent inconvenience to authors and readers.

